# Self-medication with psychedelics: a scoping review and narrative synthesis of review-level evidence

**DOI:** 10.1016/j.rcsop.2026.100709

**Published:** 2026-01-26

**Authors:** Shreya Shiju, Rohan Tirumala, Elliot Marseille

**Affiliations:** a University of California, Berkeley, CA, United States; b UC Berkeley, Collaborative for the Economics of Psychedelics (CEP), Berkeley, CA, United States

**Keywords:** Psychedelics, Self-medication, Psilocybin, LSD, Cluster headache, Microdosing, Harm reduction

## Abstract

**Background:**

As public and scientific interest in psychedelics grows, unsupervised use for health purposes is increasing. In the U.S., past-year hallucinogen use nearly doubled from 2015 to 2023. Many individuals report self-treating physical or psychological symptoms without medical supervision using psychedelics—a practice termed self-medication. Despite this trend, review-level syntheses remain scarce.

**Aim:**

This scoping review aimed to map and synthesize review-level evidence on the self-medication of psychedelics, including which substances are used, for what health-related purposes, and what benefits and harms have been reported.

**Methods:**

We conducted a scoping review of review-level evidence on self-medication with psychedelics, following the PRISMA PRISMA-ScR (2018) checklist. Searches in PubMed, PsycINFO, and Google Scholar (October–November 2024) used the terms (“self-medication” OR “self-treatment”) AND “psychedelics.” Eligible reviews examined unsupervised use of classical or non-classical psychedelics for physical, mental, or behavioral conditions. Four reviewers independently screened all records. Data extraction was conducted using Elicit AI and was manually verified by reviewers. Methodological quality was assessed using AMSTAR criteria.

**Results:**

Three reviews met inclusion criteria (systematic, scoping, narrative). Psilocybin and LSD were most frequently reported, primarily for cluster headache and chronic pain. Outcomes included abortive relief, prophylactic relief, and prolonged remission, often from microdosed regimens. Approximately 40% achieved full remission; 70% reported preventive benefit. Adverse effects were rare and brief. Motivations for self-use centered on coping, desperation, and dissatisfaction with conventional care.

**Conclusions:**

Preliminary review-level evidence suggests that individuals self-medicating with psychedelics—particularly psilocybin and LSD—report symptom relief for conditions such as cluster headache, though findings remain limited by scarce and heterogeneous data. More rigorous research is needed to clarify effectiveness, safety, and real-world patterns of use.

## 1 Background

With psychedelics gaining increasing cultural and scientific attention, public perceptions and patterns of use are shifting rapidly. National data from the Substance Abuse and Mental Health Services Administration (SAMHSA) show that past-year hallucinogen use among individuals aged 12 and older in the United States has nearly doubled from approximately 4.7 million people in 2015 to 8.5 million in 2022, and 8.8 million in 2023.1., 2. This upward trend underscores a growing public engagement with psychedelics amid renewed interest in their potential psychological and clinical benefits.

While psychedelics are often sought for spirituality, self-exploration, or enhancement of well-being, an increasing number of individuals report using them specifically to address mental or physical health symptoms outside of medical supervision. This pattern aligns with the concept of self-medication, which has become a central topic in discussions of unsupervised psychedelic use.^3^ Self-medication is defined generally as the “use of drugs to treat self-diagnosed disorders or symptoms”.^4^ Another definition, which aligns more closely with the purposes of this review, emphasizes its broader motivational basis: the use of substances to alleviate or manage perceived negative internal states, psychological or physical, such as emotional distress, anxiety, pain, or discomfort, without professional oversight (adapted from^5^). Within the psychedelic context, this may include the unsupervised use of psilocybin, LSD, ayahuasca, or other hallucinogens to self-treat mood disturbances or enhance daily functioning.^3^

Despite increasing research on psychedelics, the role of self-medication, its implications, outcomes, and risks, remains insufficiently clarified. To address this gap, we conducted a scoping review of the current review literature to summarize the current state of knowledge. Our primary aim is to identify which psychedelics are being used for self-medication and for what conditions, as well as to examine the evidence regarding associated benefits and harms.

## 2 Methods

### 2.1 Search strategy

We conducted a search for review papers on self-medication with psychedelics. Searches were conducted in PubMed and PsycINFO, with Google Scholar used as a supplementary source to ensure comprehensive retrieval. We applied the search strings (“self-medication” AND “psychedelics”) and (“self-treatment” AND “psychedelics”), after iterative refinement with the assistance of a UC Berkeley librarian to maximize retrieval of high-quality sources. Searches were conducted between October and November 2024. Searches were intentionally broad and inclusive, encompassing any population type, any psychedelic substance, and any reported physical, mental, or behavioral outcome. No citation tracking or hand-searching of reference lists was performed beyond the structured database searches. Eligible papers included reviews that examined the self-directed or unsupervised use of psychedelics for therapeutic or symptom-management purposes. The reviews covered both classical psychedelics (lysergic acid diethylamide [LSD], psilocybin, *N*,*N*-dimethyltryptamine [DMT], and mescaline) and non-classical psychedelics (3,4-methylenedioxymethamphetamine [MDMA], ketamine, ibogaine, salvinorin A, and nitrous oxide). We limited our selection to papers published in English or with an available English translation. Reviews were excluded if they focused exclusively on clinically administered psychedelic-assisted therapy or on non-therapeutic motives such as personal exploration or spirituality.

Initially, we aimed to conduct a full umbrella review; however, the limited and heterogeneous nature of the available literature did not meet the minimum requirements for homogeneity in scope and method required for that design. As a result, we adapted our protocol to perform a scoping review. We adhered to the PRISMA-ScR (2018) 22-item checklist, the established PRISMA extension for scoping reviews.

No formal review protocol was developed or registered for this scoping review. Consistent with PRISMA-ScR (2018) recommendations, all methodological decisions—including eligibility criteria, search strategy, and data-charting procedures—were established prior to data extraction but were not recorded in a publicly accessible protocol.

**Fig. 1:**
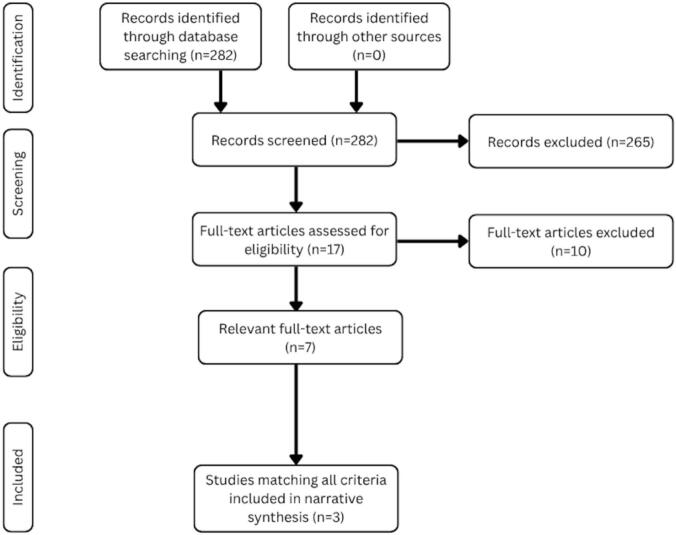
PRISMA flow diagram of review selection process.

Four reviewers independently screened titles and abstracts, with divergent judgments resolved through full-text analysis and consensus among all four reviewers. A total of 282 records were retrieved through database searches, of which 265 were excluded after screening; 17 full-text articles were assessed for eligibility, and three met all inclusion criteria for the final synthesis. These three relevant review papers — one scoping, one systematic, and one narrative review — form the core of this scoping review (see Fig. 1). Data extraction was conducted using Elicit AI, an AI-based research assistant, with both preset categories, such as *Population Characteristics*, *Quantitative Health Results*, *Qualitative Health Results*, and additional customizable extraction categories (see Table 1, Table 2, Table 3 for the complete list of categories). These automated extractions underwent manual verification by at least one reviewer per paper.

**Table 1:** Summary of recent reviews examining the therapeutic use and motivations for serotonergic psychedelics. The table outlines study authors, publication details, databases searched, and key review characteristics, including inclusion criteria, scope, and methodological approach.

Authors	Title and DOI	Journal	Publication year	Databases searched	Review Characteristics
Basedow, Lukas A. Kuitunen-Paul, Sören	Motives for the use of serotonergic psychedelics: A systematic review https://doi.org/10.1111/dar.13480	Drug and Alcohol Review	2022	– Medline, Web of Science, Embase	– Systematic review of 37 studies on motives for serotonergic psychedelic use across clinical and non-clinical populations. – Integrates quantitative (survey) and qualitative (interview/ethnographic) evidence to map motive categories.
Akash Goel, Yeshith Rai, Shayan Sivadas, Calvin Diep, Hance Clarke, Harsha Shanthanna, Karim S Ladha, Peter Nagele	Use of Psychedelics for Pain: A Scoping Review https://doi.org/10.1097/ALN.0000000000004673	Anesthesiology	2022	Ovid Medline, Cochrane, PsycINFO, EMBASE (1946–2022).	– Scoping review of human studies on psychedelics for pain (acute, chronic, cancer, headache, neuropathic). - 21 articles included (9 surveys, 7 casereports/series, 5 trials). - Inclusion criteria: human studies on psychedelics for acute, subacute, acute-on-chronic, and chronic pain; adults ≥18 years; original data; short- and long-term effects. - Only English articles included.
Nina I. Kooijman, Tim Willegers, Anke Reuser, Wim M. Mulleners, Cornelis Kramers, Kris C. P. Vissers, Selina E. I. van der Wal	Are psychedelics the answer to chronic pain: A review of current literature https://doi.org/10.1111/papr.13203	Pain Practice	2023	- Database: PubMed (search strategy developed with librarian, Radboud University).	- Narrative review - Literature up to Feb 2022 - No geographic data - Number of primary studies not stated

**Table 2:** Overview of psychedelic substances and their reported applications in pain-related conditions. The table outlines substance-specific information (compound type, dosage range, and classification) and indicates the symptoms or disorders (pain, headache, fibromyalgia) for which participants reportedly self-medicated.

Authors	Title and DOI	Substance-Specific Information	Symptoms/Disorders for Which Participants Self-Medicated, and Psychedelic Substance Used for Each		
			Pain / Chronic Pain	Headache / Migraine	Fibromyalgia
Basedow, Lukas A. Kuitunen-Paul, Sören	Motives for the use of serotonergic psychedelics: A systematic review https://doi.org/10.1111/dar.13480	– Serotonergic psychedelics: psilocybin, LSD, DMT/ayahuasca, mescaline; grouped broadly into tryptamines vs phenethylamines. - Review did not report specific dosage ranges or use frequency. - Microdosing noted but not analyzed in depth.	Not reported	Not reported	Not reported
Akash Goel, Yeshith Rai, Shayan Sivadas, Calvin Diep, Hance Clarke, Harsha Shanthanna, Karim S Ladha, Peter Nagele	Use of Psychedelics for Pain: A Scoping Review https://doi.org/10.1097/ALN.0000000000004673	– LSD, psilocybin, DMT/ayahuasca, MDMA (ketamine excluded by design). – Dosing/routes varied (oral most common; some inhaled) – LSD (5–200 μg), psilocybin (0.5 g–2–3 g), MDMA (50–150 mg).	LSD, Psilocybin, DMT	Psilocybin, LSD, DMT	Psilocybin, LSD, DMT
Nina I. Kooijman, Tim Willegers, Anke Reuser, Wim M. Mulleners, Cornelis Kramers, Kris C. P. Vissers, Selina E. I. van der Wal	Are psychedelics the answer to chronic pain: A review of current literature https://doi.org/10.1111/papr.13203	- Substances: LSD (synthetic), psilocybin (natural), ayahuasca (natural). - Classification: classic hallucinogens, serotonin-2 A receptor agonists. Dosages: LSD: 25–50 μg daily (phantom limb pain); 200–500 μg in psychotherapy; 50–200 μg recreationally. Psilocybin: 4–10 mg therapeutic effects; 10–50 mg recreational effects. - Frequency: single doses can induce remission; sub-hallucinogenic doses noted. - Microdosing: mentioned but not the focus. - Sourcing/procurement not discussed.	Psilocybin, LSD, Ayahuasca	Psilocybin, LSD	Psilocybin

**Table 3:** Summary of outcomes and participant characteristics across reviews of psychedelic use for pain management. The table details self-medication practices, outcome assessment methods, reported risk factors or cases of ineffectiveness, population demographics, and both quantitative and qualitative health results, emphasizing trends in efficacy, tolerability, and participant-reported experiences.

Authors	Title and DOI	Self-Medication Practices	Outcomes Assessment	Risk Factors/Cases of Ineffectiveness	Population Demographics	Quantitative Health Results	Qualitative Health Results
Basedow, Lukas A. Kuitunen-Paul, Sören	Motives for the use of serotonergic psychedelics: A systematic review https://doi.org/10.1111/dar.13480	– Not discussed as a primary focus; mentions of symptom relief appear in some studies without systematic tracking or supervision details.	Not applicable (the paper is a systematic review of motives for SP use, not an outcomes assessment study)	Not reported	– 17 quantitative studies (n = 11,452): community samples incl. Festivalgoers, college students, novel psychoactive substance (NPS) users. – 20 qualitative studies (*n* = 1372): psychiatric patients, LSD/ayahuasca users, microdosers.	– Across 37 studies, self-exploration was the most frequently cited motive (≈ 89% of studies), followed by coping (≈ 68%) and spiritual/mystical motives (≈ 65%). – Coping motives appeared more often in qualitative than quantitative work (75% vs 59%), though these differences were not statistically significant (all OR *p* > 0.05). – No significant associations were found between year of publication and motive frequencies.	Not reported
Akash Goel, Yeshith Rai, Shayan Sivadas, Calvin Diep, Hance Clarke, Harsha Shanthanna, Karim S Ladha, Peter Nagele	Use of Psychedelics for Pain: A Scoping Review https://doi.org/10.1097/ALN.0000000000004673	- Not the paper's main focus; does not provide detailed info on self-medication contexts. - Notes that some included studies reported on participants self-medicating.	- Primary outcomes: pain relief and reduction in pain intensity. - Secondary outcomes: side effects (mild, transient), longevity of effects, some sustained relief reported. - No detailed reporting on integration, adherence, or structured treatment practices. - Overall: psychedelics were well-tolerated, with no serious adverse events reported.	- Bonnelle et al., (2022): 13.2% (33/250) reported worsened pain — 5.3% of microdosers (*n* = 187) and 19.5% of macrodosers (*n* = 118). - Glynos et al., (2022): 1/12 fibromyalgia users saw no improvement. - Bornemann et al. (2021): 2/11 chronic pain users reported pain amplification at moderate/high doses. - Di Lorenzo et al. (2016): 4/18 cluster-headache patients found psychedelics ineffective.de Coo et al.^16^: MDMA increased cluster-headache duration; psilocybin reduced attack frequency/duration (n = 643).	- 21 studies included: 9 population surveys, 7 case studies, 5 clinical trials. - Survey populations: 10–600 participants, chronic pain & migraine focus. - Case studies: mostly <10 participants (e.g., phantom limb, chronic pain, one with 53 participants). - Clinical trials: 10–72 participants, included healthy patients, cancer patients, etc.-	– 8 studies included with self-medication data (*n* > 1847 total). – CH survey (*n* = 496): psilocybin/LSD rated comparable or superior to oxygen/triptans; preventive benefit reported in ∼70%, full remission in ∼40%. – Bonnelle et al. (*n* = 250): 147/250 micro- or macrodosed for pain; 127/187 microdosers and 118/163 macrodosers improved. – Glynos et al. (*n* = 354 fibromyalgia): 12 self-medicated; 11/12 improved. – 13.2% (33/250) reported worsened pain; MDMA extended CH periods	– Psychedelics viewed as effective or last-resort options after conventional treatment failure. – Reports describe rapid relief (within ∼20 min) for headache attacks. – Users preferred microdosing for tolerability and mood enhancement.
Nina I. Kooijman, Tim Willegers, Anke Reuser, Wim M. Mulleners, Cornelis Kramers, Kris C. P. Vissers, Selina E. I. van der Wal	Are psychedelics the answer to chronic pain: A review of current literature https://doi.org/10.1111/papr.13203	Cluster headache patients self-medicating with psilocybin/LSD to abort attacks and extend remission periods. - Some patients used psychedelics for therapeutic effect rather than recreation. - Others reported resorting to psychedelics after dissatisfaction with conventional treatments. - Psychedelics sometimes used atsub-hallucinogenic doses to avoid full psychoactive effects, reflecting desperation for relief.	- Primary outcomes: pain reduction, relief from emotional distress. - Secondary outcomes: mood changes, altered attitudes toward death. - Reported adverse effects: dizziness, nausea, blurred vision, increased heart rate/blood pressure, panic attacks, anxiety, paranoia. - Duration of effects: weeks to months. - No reporting on integration practices or treatment adherence.-	- Occasional reports of treatment ineffectiveness in chronic pain and cluster-headache self-medication samples. - MDMA sometimes associated with prolonged cluster periods or worsening symptoms. - Limited detail on adverse outcomes due to small, self-reported samples and overlapping data with Goel et al.^7^	– Adult human participants across surveys, case series/reports, and small trials; settings included headache populations and mixed chronic pain cohorts. (Demographic granularity varied by study; not uniformly reported.)	– Sewell et al.: 2/2 LSD users reported complete CH remission after a single sub-hallucinogenic dose. – Overlap with Goel et al. for CH survey data (n = 496)	– Pain cohorts include CH, migraine, fibromyalgia, neuropathic pain. – Desperation and lack of treatment efficacy cited as motives. – Self-reported use of psychedelics for pain management as “last resort.” – Italian CH survey (*n* = 54): use primarily therapeutic, not recreational. - Prophylactic effects sustained even when used infrequently and at sub-hallucinogenic doses. - Microdosing preferred by some to reduce interference with daily responsibilities, and for perceived benefits to mood/creativity. – LSD and psilocybin associated with both abortive and preventive effects. – MDMA and DMT less effective; MDMA prolongedattacks in some cases.

Although critical appraisal of included sources is not required for scoping reviews, we judged it important given the very small number of available reviews. We therefore applied the AMSTAR-2 tool to each included review to better understand the methodological strengths and limitations of the evidence base. Our intention was descriptive rather than exclusionary: no reviews were excluded on the basis of AMSTAR-2, and we did not calculate summary scores. Instead, AMSTAR-2 items were used to contextualize the credibility of each review's findings and to inform the interpretation of our narrative synthesis.

## 3 Results

### 3.1 General summary of included review articles

The review literature directly examining self-medication with psychedelics is extremely limited. Even among the three reviews we identified that touch on this topic, self-medication typically appears as a secondary topic rather than a primary focus. We provide a brief overview of each of these three reviews here:

The first, *Motives for the Use of Serotonergic Psychedelics: A Systematic Review,*^6^ sought to synthesize existing evidence on why individuals use serotonergic psychedelics. Drawing from 37 studies across diverse populations (including 17 quantitative studies, *n* = 11,452 participants, and 20 qualitative studies, *n* > 1372), the authors identified and categorized the principal motives for psychedelic use, classifying them as awareness, coping, and enhancement. Although several included studies referenced coping or symptom-management behaviors, the review itself did not explicitly center on self-medication, instead situating such motives within broader psychological and experiential frameworks.

The second review, *Use of Psychedelics for Pain: A Scoping Review,*^7^ examined the emerging evidence on psychedelic compounds in the management of pain conditions such as migraines and cluster headaches. This work collated both clinical and naturalistic studies, some of which included self-directed or unsupervised use of psychedelics for analgesic purposes. Among these, four studies involving self-medication for headache-related conditions (*n* > 1207; one study did not specify n) and four studies involving patients with other conditions who self-medicated (*n* = 640) were identified. However, self-medication was not an analytical focus, appearing mainly in the context of symptom relief reports within clinical settings for psychedelic administration.

Finally, *Are Psychedelics the Answer to Chronic Pain: A Review of Current Literature*^8^ explored the potential role of psychedelics in chronic and recurrent pain disorders. The review discussed both clinical findings and patient-reported experiences, noting self-reported instances of individuals using psychedelics to self-manage pain. As with the other two papers, these examples were referenced to illustrate broader therapeutic interest rather than to systematically evaluate self-medicative behavior.

### 3.2 Psychedelics substances used for self-medication

Across the included reviews, the substances most frequently discussed in self-medication contexts are psilocybin and LSD, with MDMA and DMT appearing less often and with less consistent therapeutic signal for pain relief.^6^, ^7^, ^8^ See Table 2.

### 3.3 Motives for self-medication

Across samples, coping motives, defined as using psychedelics to reduce negative affective states, were reported in 67% of the 37 studies included in Basedow and Kuitunen-Paul.^6^ In addition, coping was listed as a motive in five studies with samples consisting of patients of varying conditions (*n* = 1255), including two studies involving psychiatric patients (*n* = 170), suggesting that self-medication may play a role in efforts to manage negative affect among individuals with physical and mental health concerns. Other motives for self-medication are evident in Kooijman et al.^8^ Qualitative accounts in pain cohorts describe being motivated by “desperation” after exhausting conventional options or experiencing inadequate relief or intolerable side effects, i.e., as a “last resort”. Consistent with this, an Italian survey of 54 cluster headache patients reported that psychedelics were rarely used for recreational purposes but primarily for their perceived effectiveness in treating the condition.^8^ Another study found that individuals who self-medicated were not interested in the psychoactive effects at all, emphasizing therapeutic intent over recreation (Andersson et al., as cited in^8^).

### 3.4 Target conditions and use cases

Among the three papers we identified, the most prevalent clinical self-medication pattern involved primary headache disorders, especially cluster headache, with individuals using psilocybin and LSD for both acute (abortive) relief and prophylaxis.^7^, ^8^ Reports also include migraineurs experimenting with similar strategies, though relief from cluster headache is more frequently described.^8^ In Bonnelle et al. (2022, as cited in^7^), a broad range of pain conditions—including cluster headaches, fibromyalgia, migraines, tension headaches, sciatica, nerve pain, muscle pain, and arthritis—were examined in a sample of 250 participants, 147 of whom reported macro- or microdosing specifically for pain management. It also included a study by Glynos et al. (2022, as cited in^7^), with 354 fibromyalgia patients, of which 12 were using psychedelics to self-medicate for chronic pain.^7^ While the broader motive review points to coping with mood and anxiety symptoms as common reasons to use psychedelics, systematic, diagnosis-specific evidence outside headache conditions is comparatively sparse in the self-medication literature.^6^

### 3.5 Dosing patterns (micro- vs. macro-dosing) and number of doses

Dosing details are inconsistently reported in self-medication contexts: for example, six of the eight relevant individual studies included in Goel et al.^7^ did not provide precise dosing information, whereas others explicitly described single-dose “macrodosing” or repeated sub-hallucinogenic “microdosing.” In cluster headache, individuals have reported that a single dose of LSD can be sufficient to induce remission. The full psychoactive experience may not be necessary to achieve therapeutic benefit, as symptom relief was often reported even at sub-hallucinogenic levels. Microdosing was often preferred to minimize psychoactive disruption and because users perceived mood and creativity benefits alongside headache relief.^8^ In some cases, symptom relief from abortive psilocybin or LSD was rapid (within ∼20 min), highlighting a time-sensitive rationale for self-administration during attacks.^7^

### 3.6 Comparative efficacy vs. conventional therapies

In a cluster headache self-report survey of *n* = 496, individuals who self-medicated with indoleamine hallucinogens, including LSD and psilocybin, described using them as both abortive and preventive agents.^7^ As abortive treatments, these psychedelics were reported to be comparable to traditional cluster headache therapies like high-flow oxygen and, in some cases, more effective than intranasal or oral triptans.^7^ As preventive agents, psilocybin and LSD were described as reducing attack frequency and intensity, sometimes after a single, sub-hallucinogenic administration.^7^ Their preventive effects were further reported as more effective than traditional first-line treatments such as verapamil and prednisone.^7^ Moreover, the survey data indicate (n = 496) that the abortive effectiveness of psilocybin and LSD was rated as complete in roughly one-third of attempts and moderate in about two-thirds, while preventive use yielded moderate protection in approximately 70% and complete remission in about 40% of cases.^7^, ^8^

Goel et al. also discussed positive findings in two non-cluster headache contexts. In the previously discussed study by Bonnelle et al. (2022, as cited in^7^), most participants reported pain relief: among microdosers, 127 out of 187 experienced reductions in pain symptoms, and among macrodosers, 118 out of 163 reported similar improvements. Likewise, in Glynos et al. (2022, as cited in^7^), 11 of 12 fibromyalgia patients self-medicating with psychedelics for chronic pain experienced symptom improvements.

### 3.7 Comparative efficacy across psychedelic substances

Among substances used for headache self-medication, psilocybin and LSD most consistently appear in accounts of both acute abortion and prophylaxis, with some users reporting prolonged remission unique to these agents.^7^, ^8^ LSD was found to exhibit even stronger potential for treating cluster headache than psilocybin.^7^ In contrast, MDMA and DMT are used less commonly and show inconsistent pain-related profiles in the self-medication context: MDMA appeared to worsen condition in certain cases, whereas psilocybin was associated with reductions in both attack frequency and duration, based on reports from a total sample of 643 participants. DMT was not shown to have the same remission results as LSD and psilocybin. These differential patterns suggest that classic serotonergic psychedelics (psilocybin/LSD) may hold more perceived utility for headache self-management than MDMA or DMT, at least within existing self-report datasets.^7^, ^8^

### 3.8 Cases of ineffectiveness

Reports of ineffectiveness or symptom worsening were relatively rare but present across several self-medication studies highlighted in Goel et al.^7^ For example, in Bonnelle et al. (2022, as cited in^7^), 33 out of 250 participants (13.2%) reported an increase in pain symptoms, including 5.3% of microdosers (*n* = 187) and 19.5% of macrodosers (*n* = 118). In Glynos et al. (2022, as cited in^7^), 1 of 12 fibromyalgia patients using psychedelics for chronic pain did not experience symptom improvement, while in Bornemann et al. (2021, as cited in^7^), 2 of 11 chronic pain contributors reported that psychedelic agents amplified pain at moderate or high doses. Similarly, in the cluster headache study by Di Lorenzo et al. (2016, as cited in^7^), 4 of 18 self-medicating cluster headache patients found the treatment ineffective. Substance-specific side effects were also noted: in de Coo et al.,^16^ MDMA was reported to increase the duration of cluster headache attacks.

### 3.9 Evidence quality and overlap

Two pain-focused reviews summarize overlapping primary sources (e.g., survey data on psilocybin/LSD for cluster headache), which convergently report acute abortion, prophylaxis, and remission extension with classic psychedelics.^7^, ^8^ Both emphasize limitations: small samples, reliance on retrospective self-report, variable or missing dose information, and potential selection biases toward users with positive experiences.^7^, ^8^ The motives review, while broader and not limited to pain, similarly synthesizes heterogeneous naturalistic samples and underscores that coping motives are widespread but not diagnostic-specific.^6^

### 3.10 AMSTAR guidelines

The two articles by Basedow & Kuitunen-Paul^6^ and Goel et al.^7^ each met six of the eleven AMSTAR criteria, indicating moderate methodological quality, while the review by Kooijman et al.^8^ met four of the eleven, suggesting low quality. The two moderate-quality reviews were most consistent in fulfilling domains related to duplicate study selection and data extraction (*AMSTAR 2*), reporting characteristics of included studies (*AMSTAR 6)*, applying appropriate synthesis methods (*AMSTAR 9*), and disclosing conflicts of interest (*AMSTAR 11*). However, across all three reviews, the most frequently lacking domains were the use of an a priori design (*AMSTAR 1*), inclusion of grey literature (*AMSTAR 4*), assessment of study quality (*AMSTAR 7*), use of study quality in formulating conclusions (*AMSTAR 8*), and assessment of publication bias (*AMSTAR 10*). The absence of these key domains suggests that while the reviews maintained transparency in reporting and data handling, they exhibited consistent weaknesses in methodological rigor and bias control. Collectively, this pattern reflects a developing but still exploratory stage of evidence synthesis in the literature on psychedelics and pain, limiting the overall reliability and generalizability of their conclusions. See Table 4.

**Table 4:** Methodological quality assessment of included reviews using the AMSTAR (A Measurement Tool to Assess Systematic Reviews) criteria. The table summarizes compliance with 11 AMSTAR domains, evaluating aspects such as protocol transparency, literature search rigor, data extraction methods, assessment of study quality, and reporting of conflicts of interest.

Authors	Title and DOI	AMSTAR 1 -Was an ‘a priori’ design provided?	AMSTAR 2 - Was there duplicate study selection and data extraction?	AMSTAR 3 - Was a comprehensive literature search performed?	AMSTAR 4 - Was the status of publication (e. g., grey literature) used as an inclusion criterion?	AMSTAR 5 -Was a list of studies (included and excluded) provided?	AMSTAR 6 - Were the characteristics of the included studies provided?	AMSTAR 7 - Was the scientific quality of the included studies assessed and documented?	AMSTAR 8 - Was the scientific quality of the included studies used appropriately in formulating conclusions?	AMSTAR 9 - Were the methods used to combine the findings of studies appropriate?	AMSTAR 10 - Was the likelihood of publication bias assessed?	AMSTAR 11 - Was the conflict of interest included?
Basedow, Lukas A. Kuitunen-Paul, Sören	Motives for the use of serotonergic psychedelics: A systematic review https://doi.org/10.1111/dar.13480	NO	YES	YES	NO	YES	YES	NO	NO	YES	NO	YES
Akash Goel, Yeshith Rai, Shayan Sivadas, Calvin Diep, Hance Clarke, Harsha Shanthanna, Karim S Ladha, Peter Nagele	Use of Psychedelics for Pain: A Scoping Review https://doi.org/10.1097/ALN.0000000000004673	YES	YES	YES	NO	NO	YES	NO	NO	YES	NO	YES
Nina I. Kooijman, Tim Willegers, Anke Reuser, Wim M. Mulleners, Cornelis Kramers, Kris C. P. Vissers, Selina E. I. van der Wal	Are psychedelics the answer to chronic pain: A review of current literature https://doi.org/10.1111/papr.13203	NO	YES	NO	NO	NO	YES	NO	NO	YES	NO	YES

## 4 Discussion

Reports within the included reviews describe symptom relief among individuals self-medicating for cluster headaches and chronic pain, although these findings are based primarily on self-selected, retrospective accounts and remain highly preliminary. The emerging evidence base depicts psychedelic self-medication as promising yet undercharacterized, a practice largely motivated by patient desperation and dissatisfaction with conventional therapies. Several primary studies, as summarized within the included reviews, describe rapid abortive effects, possible prophylactic benefits, or episodes of remission; however, these observations involve small sample sizes and should be interpreted cautiously.

Beyond pain-related conditions, the review by Basedow and Kuitunen-Paul^6^ illustrates that psychedelic use in naturalistic settings is frequently motivated by efforts to manage negative affect, such as distress, anxiety, or low mood. This suggests that self-medication practices may extend into mood- and anxiety-related contexts, even though the review literature we identified focused predominantly on headache disorders. Indeed, improvements in mood and emotional well-being have been reported in clinical (non–self-medication) psychedelic therapy studies involving chronic pain conditions, indicating a potential link between psychedelic use and affective outcomes that warrants further investigation in self-medication contexts as well.^7^

A key observation across studies is that therapeutic benefit often occurred independently of the full psychedelic or hallucinogenic experience. Several reports noted symptom improvement at sub-hallucinogenic or microdosed levels, reinforcing the idea that the psychoactive effects of these compounds may not be necessary for analgesia or prophylaxis. This distinction challenges the traditional assumption that perceptual experiences are intrinsic to psychedelic efficacy and points instead to possible neuromodulatory, serotonergic, or anti-inflammatory mechanisms operating below perceptual thresholds.^9^, ^10^, ^11^ At the same time, microdosing was repeatedly associated with fewer side effects and better tolerability, while still yielding substantial self-reported symptom relief and mood enhancement.

The safety profile described across studies appears encouraging. Reports of adverse effects or symptom worsening were rare and mild in proportion to total samples. In the scoping review by Goel et al.,^7^ fewer than 15% of participants reported increased pain, and most adverse outcomes were transient or dose-related. Substance-specific effects were also limited: while MDMA prolonged cluster headache attacks in one participant, psilocybin and LSD were consistently associated with reduced frequency, duration, and intensity.

The predominance of cluster headache (CH) in the limited literature included in this review may reflect both the debilitating severity and treatment resistance of this condition. CH is sometimes referred to as the “suicide headache,” with lifetime suicide attempt rates estimated to be up to 20 times higher than the general population and nearly 55% of patients reporting suicidal ideation during active periods.^12^ In this context, the willingness of individuals to experiment with unsupervised psychedelics underscores both the desperation for effective relief and the perceived therapeutic credibility these substances hold among patients failed by conventional care. However, a broader evidence base than that which currently exists would be needed to establish the relative prevalence of CH as a motive for psychedelic self-medication relative to other conditions.

It is also important to note that many of the psychedelic substances discussed in the included reviews—such as psilocybin, LSD, and MDMA—are classified as controlled or Schedule I substances under U.S. federal law and international regulations. As a result, self-medication with these compounds may involve legal risks or violations, even when undertaken with therapeutic intent. The present review summarizes existing literature and does not endorse or encourage unsupervised or illegal use; rather, this context underscores the need for rigorous, ethically conducted research and for evidence-informed public health guidance as interest in these substances continues to grow.

Moreover, the current evidence base is constrained by several methodological limitations. The reliance on self-selected, retrospective, and largely unverified reports introduces bias toward positive outcomes. Few studies provided standardized dosing, objective endpoints, or prospective follow-up, limiting causal inference. Moreover, the heavy focus on headache disorders leaves major gaps in understanding how self-medication practices manifest in other conditions such as depression, PTSD, or chronic neuropathic pain. Notably, none of the reviewed studies discussed self-medication in the context of tobacco, alcohol, or opioid use disorders, domains that emerging evidence identifies as among the most promising areas for psychedelic-assisted therapy.^13^, ^14^, ^15^ This omission underscores the narrow scope of current reviews and the need for broader synthesis across conditions where psychedelic interventions show therapeutic promise.

Another key limitation involves incomplete and imprecise reporting within the reviewed literature. Important details from primary studies were sometimes missing or summarized inaccurately, which can distort how both efficacy and safety are interpreted. For example, Goel et al.^7^ mentioned an MDMA-related adverse effect but did not clarify that it occurred in only one of thirty participants who had self-medicated with MDMA.^16^ Similarly, Kooijman et al.^8^ reported that a single dose of LSD appeared sufficient to induce remission in cluster headache but did not note that this conclusion was based on just two individuals who had self-medicated with sub-hallucinogenic doses, out of a larger group of twenty-one using psychedelics for chronic cluster headache.^17^ In another instance, the “within 20 minutes” rapid relief described by Goel et al. referred specifically to 17 of 20 individuals with episodic cluster headache who had self-medicated with psilocybin.^17^ Together, these examples illustrate that the reviews were not always comprehensive in their data extraction or synthesis, at times overlooking important contextual or quantitative information from the original reports.

### 4.1 Future directions

Taken together, the results indicate an urgent need for structured observational and pragmatic trials of real-world, patient-initiated psychedelic use, with rigorous outcomes and safety monitoring in cluster headache and refractory pain. Because formal access pathways remain limited and uneven, many individuals may turn to self-medication while awaiting broader legal or clinical availability. In the United States, Oregon has begun implementing a regulated psilocybin services program under the Oregon Psilocybin Services Act (2020),^19^ while Colorado has established a comparable state-sponsored system following Proposition 122 (2022), which decriminalized certain natural psychedelics and created both clinical and non-clinical pathways for supervised use.^18^ However, despite these developments, access remains geographically limited, and most patients across the country still lack participation opportunities, reflecting an ongoing regulatory lag between public demand and formal medical oversight.^20^ As awareness of the potential benefits of psychedelic self-use grows, particularly among populations facing severe symptoms and limited medical options, such as those with cluster headache, it is likely that self-medication practices will continue to expand.

Resources that provide peer support and harm reduction, such as the Fireside Project,^21^ represent important steps toward safer community-based guidance. This initiative goes beyond informal information-sharing in online support groups by offering trained peer support for individuals engaging with psychedelics. However, more structured, accessible, and medically informed resources are urgently needed to bridge the gap between emerging scientific understanding and patient-driven practice.

Finally, future research should include a comprehensive review of studies published in the past few years, as multiple recent investigations and case reports appear not to have been captured in the three major reviews examined here. Expanding the evidence base will allow for a more nuanced understanding of self-medication behaviors, dosing strategies, and safety outcomes, thereby informing both clinical trial design and public health guidance.

## CRediT authorship contribution statement

**Shreya Shiju:** Writing – review & editing, Writing – original draft, Visualization, Methodology, Formal analysis, Data curation. **Rohan Tirumala:** Writing – review & editing, Writing – original draft, Visualization, Methodology, Formal analysis, Data curation. **Elliot Marseille:** Writing – review & editing, Validation, Supervision, Project administration, Investigation, Conceptualization.

## Ethics approval

This article is based on previously conducted studies and does not contain any new studies with human participants or animals performed by any of the authors.

## Funding

No funding or sponsorship was received for this study or publication of this article.

## Declaration of competing interest

The authors declare that they have no known competing financial interests or personal relationships that could have appeared to influence the work reported in this paper.

## Data Availability

Data sharing is not applicable to this article as no datasets were generated or analyzed during the current study.
